# Biosynthetic Collagen-Analog Hydrogels Stimulate Endogenous Regrowth of Rabbit Corneas: A Pilot Study

**DOI:** 10.3390/vetsci12080785

**Published:** 2025-08-21

**Authors:** Iris Timmerman, Marie-Claude Robert, Claire Vergneau-Grosset, Tristan Juette, Javier Benito, Marta Garbin, Mostafa Zamani-Roudbaraki, Mona Moradi, Hamid Goodarzi, Christos Boutopoulos, Marie-Odile Benoit-Biancamano, May Griffith, Maria Vanore

**Affiliations:** 1 Faculty of Veterinary Medicine, Université de Montréal, 3200 Rue Sicotte, Saint-Hyacinthe, QC J2S 2M2, Canada; claire.grosset@umontreal.ca (C.V.-G.); tristan.juette@umontreal.ce (T.J.); javier.benito@umontreal.ca (J.B.); marta.garbin@umontreal.ca (M.G.); marie-odile.benoit-biancamano@umontreal.ca (M.-O.B.-B.); 2 Department of Ophthalmology, University of Montreal and Maisonneuve-Rosemont Hospital Research Centre, 5690 Boul. Rosemont, Pavillon Claudine D’Amours, Montreal, QC H1T 2H2, Canadamostafa.zamani.roudbaraki@umontreal.ca (M.Z.-R.); mona.moradi@umontreal.ca (M.M.); hamid.goodarzi@umontreal.ca (H.G.); christos.boutopoulos@umontreal.ca (C.B.); may.griffith@umontreal.ca (M.G.)

**Keywords:** corneas, collagen analogs, corneal regeneration, allografts, rabbits, anterior lamellar keratoplasty

## Abstract

Pro-regenerative corneal implants are being developed to improve corneal healing for companion animals in clinical practice. This pilot study evaluated early corneal tissue and nerve regeneration using biosynthetic collagen-analog hydrogels (CAH) in liquid and solid forms. Their efficacy was compared to each other and to allografts on nine white New Zealand rabbits, divided in three groups of three. Each rabbit cornea underwent keratectomy followed by grafting with either a control allograft cornea, liquid injectable, or solid CAH implant. Corneal healing was assessed over 16 weeks using clinical exams, esthesiometry, in vivo confocal microscopy, and optical coherence tomography. One rabbit per group was euthanized at 3, 10, and 16 weeks for histopathological analysis. Both liquid and solid implants enabled corneal re-epithelialization and regeneration of stromal tissue and corneal nerves. Esthesiometric values indicated faster nerve regeneration in rabbits grafted with biosynthetic implants compared to allografts (*p* < 0.005). By 16 weeks, regenerated neocorneas achieved transparency comparable to allografts. Solid and liquid CAH implants supported complete corneal tissue and nerve regeneration in the studied rabbits. These results suggest that with further research and development, the current gold standard for corneal transplantation could be replaced by high-performing, easily produced biosynthetic alternatives.

## Simple Summary

Preserving visual function and ocular integrity in the presence of corneal disease remains challenging due to the limitations of existing methods. The materials currently in use present several drawbacks, including reduced transparency, uncertain availability, and high complication rates. In veterinary medicine, corneal grafting in companion animals is further complicated by the limited availability of high-quality allografts, which remain the gold standard for corneal transplantation. Recently, a new type of corneal implant has been developed, composed of synthetic collagen and acellular scaffolds that stimulate host cell regeneration, thereby preventing immune rejection and promoting integration of the biomaterial into the cornea. These implants are inexpensive, non-immunogenic, and easy to manufacture, and could eventually improve the accessibility of corneal transplantation while reducing associated complication rates. The aim of our study was to expand current knowledge on these novel materials by evaluating two types of synthetic collagen in comparison with allografts. Our findings suggest that biosynthetic alternatives could represent a promising future treatment option.

## 1. Introduction

The cornea’s essential function in ensuring transparency and refracting light to the retina with minimal reflection is facilitated by its precise arrangement of collagen fibrils. Corneal injuries disrupt this structure, often leading to permanent transparency loss, which can severely impair vision or cause blindness. In veterinary medicine, the eye’s integrity and vision is often affected significantly by corneal ulcers, which are among the leading causes of ophthalmic consultations [1,2], while in human medicine, an estimated 12 to 23 million people suffer from visual impairment due to corneal conditions [3,4]. Homologous corneal grafts, or allografts, are the gold standard for optimal healing and transparency. However, they are rarely performed in veterinary medicine due to the logistical challenges of harvesting and preserving them [5], and their availability is limited in human medicine due to a severe shortage of corneal donors [6].

To address these issues, many synthetic or biosynthetic corneal substitutes have been proposed. Recent research focuses on promoting corneal tissue regeneration as an alternative to donor transplantation [7]. Extracellular matrix (ECM) polymers play a crucial role in the development of various tissues in the body and participate in wound healing and regeneration. However, irregular and disorganized ECM production that fails to recapitulate the original ordered structures can lead to scar formation [8]. Thus, biomaterials that mimic the organized matrix structure have been developed to promote normal tissue formation rather than scar tissue. For corneal regeneration, both decellularized corneal ECM and synthetic ECM analogs have been investigated [7,9]. While both approaches appear promising, synthetic analogs offer advantages, such as minimal risk of immune reactions [10], xenogeneic allergic reactions [11], or disease transmission [12].

Griffith and collaborators have pioneered the use of synthetic collagen-based materials for corneal regeneration in animal models [13] and in a first-in-human clinical trial [14]. In both contexts, recombinant human collagen type III (RHC-III) promoted the regeneration of corneal tissue and nerves. However, RHC-III is a large and complex molecule to produce and is relatively chemically inert. To address these limitations, several laboratories developed short collagen-like peptides (CLPs) as biofunctional alternatives to full-length replacements. These peptides can be easily produced on solid supports and have the ability to self-assemble into triple helical structures that resemble those of collagen [15,16]. Various synthetic polymers have been tested to stabilize the helix and make the resulting molecule biologically active. Among them, polyethylene glycol (PEG), when conjugated to CLP, produced hydrogels with suitable physical and mechanical properties. The CLP-PEG implants were sufficiently robust for implantation and supported the regeneration of corneal tissues and nerves as effectively as implants made from RHC-III implants [16]. However, although PEG is considered a relatively bioinert polymer, it has been associated with allergic reactions, most notably in COVID-19 vaccines [17,18,19]. Here, we noted its effects as a component of corneal implants.

To mitigate inflammation associated with biomaterial implantation, multiple approaches have been developed, including the integration of 2-methacryloyloxyethyl phosphorylcholine (MPC) [19,20]. We found that incorporating MPC into RHC-III corneal implants suppressed inflammation and neovascularization in rabbit models of corneal alkali burns [12], as well as in human patients with corneal pathologies and inflammation considered at high risk for rejecting conventional corneal transplants [18]. For inflamed corneas, MPC, which has known inflammation-suppressing properties, may facilitate regeneration. In previous studies, corneal hydrogel implants composed of RHC-III or CLPs combined with MPC were shown to modulate inflammation and promote stable healing [13,20,21].

In both veterinary and human patients, cyanoacrylate glue is sometimes used to seal corneal perforations, which are considered medical emergencies, as it is practical and easy to use. However, it is toxic to the cornea and often fails to prevent the need for corneal transplantation [22]. That is why a liquid version of collagen-like peptide hydrogel has been developed as an injectable sealant filler that can be used by an ophthalmologist or trained medical personnel without requiring the intervention of an experienced surgeon [23]. This liquid implant promotes corneal regeneration comparable to that of solid implants when tested in rabbits and mini-pigs [23]. Juarez et al. recently confirmed its capabilities in two cats, demonstrating significant epithelial, stromal, and nerve regeneration, as well as successful filling of a perforation [24]. However, further evidence is necessary before considering its application in clinical practice.

We previously examined the biocompatibility and efficacy of CLP-PEG-based hydrogels, both as solid prefabricated implants and as in situ gelling liquid hydrogels, in mini-pigs and cats [21,23,24,25]. These were long-term studies, and after 12 months, regenerated corneas closely resembled healthy, untreated contralateral corneas. That being said, our work provides several novel contributions. First, we directly compare the performance of these biosynthetic implants with the current gold standard (corneal allografting) in a controlled in vivo setting. We also assess both the structural and functional healing of the cornea during the early postoperative period, including corneal sensitivity and histological analysis at defined time points. Finally, our work focuses on the clinical evolution after implantation and aims to provide translational data to guide the design of future clinical trials.

In this study, we compared early responses to pre-polymerized and in situ polymerized hydrogels to allografts in rabbits and their effectiveness in recreating a cornea resembling a native cornea during the early stages of healing. To do so, the corneal healing process was monitored over time to observe the stages of regeneration. We assessed morphological and functional corneal regeneration, biocompatibility, and transparency post-corneal grafting.

## 2. Materials and Methods

### 2.1. Preparation of Solid CAH Implants

Solid collagen analogs of CLPs were fabricated into hydrogel implants measuring 10 mm in diameter and 350 µm in thickness. Solid CAH implants were prepared following the protocols for CLP-PEG-MPC hydrogels as described by Simpson et al. [21]. Specifically, thinner molds were employed, and the water content was reduced by 50%, resulting in implants with over 15% solids, consistent with the solid content found in rabbits [26]. The CLP-PEG-MPC was crosslinked using 4-(4,6-Dimethoxy-1,3,5-triazin-2-yl)-4-methylmorpholinium chloride (DMTMM; Sigma Aldrich, Oakville, Canada) and then dispensed into polypropylene molds for curing. Following this process, the cornea-shaped implants were stored in 0.1 M phosphate-buffered saline (PBS) until required for use. To maintain sterility, 1% chloroform (*v*/*v*) was added.

### 2.2. Preparation of Liquid CAH Implants

Liquid CAH implants were prepared by conjugating CLP to poly MPC with a PEG template. This proprietary formulation is the subject of a pending PCT patent (PCT #CA2024050799, filed on 2024-06-13). The CLP-PEG-MPC, along with the DMTMM crosslinker, was injected into the wound bed using a double-barreled syringe-like device and allowed to gel in situ.

### 2.3. Preparation of Corneal Allografts

Corneal allografts were obtained following lamellar keratectomy of rabbit eyes receiving solid or liquid implants. Stromal dissection at approximately 60% depth was performed using a crescent blade. The isolated corneal tissue was preserved in PBS and refrigerated until implantation in another animal.

### 2.4. Animal Study Design

#### Animal Source and Ethical Approval

Nine 4-month-old female white New Zealand rabbits (SPF colony) with a mean weight of 2.5 ± 0.2 kg were enrolled in this study. All procedures were conducted in accordance with the Use of Animals in Ophthalmic and Vision Research statement of the Association for Research in Vision and Ophthalmology (ARVO) and the Guidelines for Ethical Conduct in the Care and Use of Animals in Research. The study was approved by the Animal Care and Use Committee (Comité d’éthique de l’utilisation des animaux, CÉUA 23-Rech-2257).

The rabbits were housed in the Division Ferme et Animaleries (FANI) in individual stainless-steel cages (69 × 69 × 45 cm), with a cyclic rotation in floor cages (130 × 150 × 100 cm) and maintained in an open system. The room temperature (22 ± 2 °C), humidity (63 ± 3%), and animal husbandry were standardized throughout the experiment. The experiments were conducted between 8:00 a.m. and 5:00 p.m.

### 2.5. Preoperative Evaluation

#### 2.5.1. Clinical Evaluation

Upon arrival, rabbits were acclimatized and conditioned for 10 days to allow physiological adjustment to the environmental changes before beginning experiments. They were randomly assigned to one of three groups: allograft (rabbits 3, 8, 9), solid CAH implants (rabbits 1, 2, 7), and liquid CAH implants (rabbits 4, 5, 6). No data were excluded from the study.

Blood samples were collected, including hematological and biochemical analyses, all of which showed no abnormalities. One week before surgery, comprehensive ophthalmic examinations were performed, including slit-lamp evaluation, intraocular pressure measurement (TONOVET Plus, Icare Finland Oy, Helsinki, Finland), Schirmer test, and fluorescein staining. Corneal transparency, neovascularization, conjunctival congestion, chemosis, ocular discharge, Tyndall effect, and fluorescein staining were scored using the modified McDonald–Shadduck scale [27]. This scoring system facilitated the objective and reproducible quantification of these parameters throughout the study follow-up period, ensuring consistency and reliability in the assessment.

#### 2.5.2. Esthesiometry

Esthesiometric testing was performed using the Cochet–Bonnet esthesiometer (Luneau Technology, Pont-de-l’Arche, France) to assess corneal sensitivity. This instrument measures mechanical sensitivity by eliciting the corneal reflex: a thin nylon filament is applied perpendicularly to the cornea until it bends slightly, triggering a blink response if the stimulus is perceived. The pressure exerted on the cornea is inversely proportional to the filament length, with longer lengths indicating greater corneal sensitivity [28]. Eyelashes were trimmed to prevent interference. The nylon filament was applied to the cornea, starting at its maximum length (6 cm), and shortened in 0.5 cm increments until a blink response was observed. Only central measurements were performed.

#### 2.5.3. Imaging

All rabbits were sedated using an intramuscular combination of ketamine (5–6 mg/kg; Narketan^®^, Vetoquinol N.-A. Inc., Lavaltrie, QC, Canada), midazolam (0.5 mg/kg; Dormazolam^®^, Dechra Veterinary Products Inc., Pointe-Claire, QC, Canada), dexmedetomidine (0.025 mg/kg; Dexdomitor^®^, Zoetis Canada Inc, Kirkland, QC, Canada), and butorphanol (0.5 mg/kg; Dolorex^®^, MERCK ANIMAL HEALTH Intervet Inc., Whitehouse Station, NJ, USA). The rabbits were monitored during sedation using a pulse oximeter (Masimo 9847 Rad-G Continuous Pulse Oximeter; Masimo Corporation, Irvine, CA, USA) to assess their pulse rate and arterial oxygen saturation.

Under sedation, corneal evaluations were performed using optical coherence tomography (OCT SPECTRALIS, Heidelberg Engineering GmbH, Heidelberg, Germany) and confocal microscopy (Confoscan 3, Nidek Technologies, San Jose, CA, USA). Full thickness confocal microscopy (ICVM) was performed in the center of the cornea and used to evaluate corneal morphology, including corneal layers, keratocytes, and nerve growth.

### 2.6. Surgical Procedure

#### 2.6.1. Anesthesia and Preparation

One week after the initial eye evaluations, corneal surgeries were performed under general anesthesia by two experienced surgeons (MVA, MCR). Chemical immobilization was initiated with intramuscular premedication comprising midazolam (0.5 mg/kg; Dormazolam^®^, Dechra Veterinary Products Inc., Pointe-Claire, QC, Canada), ketamine (5 mg/kg; Narketan^®^, Vetoquinol N.-A. Inc., Lavaltrie, QC, Canada), and hydromorphone (0.1 mg/kg; HYDROmorphone HP^®^10, Sandoz, Saint-Hubert, QC, Canada). Anesthesia was induced via intravenous injection of propofol (PropoFlo 28^®^, Zoetis Canada Inc., QC, Kirkland, Canada) and maintained with isoflurane in 100% oxygen, delivered via an endotracheal tube (internal diameter 2.5 or 3 mm) connected to a non-rebreathing system (Bain circuit). Rabbits were mechanically ventilated throughout the procedure. Just before the first surgical incision, 4 to 6 drops of bupivacaine (Bupivacaine 0.5%, Sterimax Inc., Oakville, ON, Canada) were applied to the corneal surface for local anesthesia.

#### 2.6.2. Surgical Technique

The surgeries, conducted in week 0, involved operating on the right eye while the left eye served as a control. A corneal trephine with vacuum (Recipient Vacuum Trephine, CorneaGen, Seattle, WA, USA) was used to create a 6.0 mm diameter incision. The depth of the incision was controlled by the number of turns of the trephine head, with each turn corresponding to a calibrated tissue depth, allowing consistent excisions to approximately 300 µm. The corneal flap was dissected with a crescent knife (Sharpoint Clear Corneal Knives, Grayline Medical, Norwalk, CA, USA) and excised. Each rabbit received either solid collagen-analog hydrogel implants, allografts, or liquid collagen-analog hydrogel implants placed in the keratectomy bed corresponding to the randomly assigned group.

Solid CAH implants were trephined to a diameter of 6.5 mm before placing into the keratectomy bed and suturing. The slightly larger diameter of the hydrogel ensured a snug fit into the wound bed. The allografts were 6.0 mm in diameter as they were prepared from excised buttons on previously operated rabbits. For the solid CAH implants and allografts, the graft was anchored with three mattress sutures using 10/0 Nylon (Ethilon Suture, Grayline Medical, Norwalk, CA, USA), placed 1mm from the edge of the keratectomy. The suture knots were buried into the host stroma. For liquid CAH implants, the liquid hydrogel was injected into the keratectomy bed using a specially designed 3D-printed syringe and allowed to solidify for 30 min without irrigation. A Canis II corneal lens (Vetlexicon, Fort Mill, SC, USA) was placed under the nictitating membrane, and temporary blepharorrhaphy was performed with a U-shaped suture on the lateral part of the eyelids to ensure the protection of the ocular surface (silicone tubing and 5/0 Prolene (Prolene Monofilament Suture, Grayline Medical, Norwalk, CA, USA).

#### 2.6.3. Postoperative Care

Postoperative analgesia included meloxicam (1 mg/kg per os once daily for 5 days; Metacam^®^, Boehringer Ingelhein Animal Health Canada Inc., Burlington, ON, Canada), and long-acting buprenorphine (120 µg/kg subcutaneous every 72 h for 7 days; Buprenol, Labiana Life Sciences S.A. Terrasa, Barcelona, Spain). Pain was assessed twice a day for one week with the Bristol Pain Scale [29], and short-acting buprenorphine (0.05 mg/kg subcutaneous; Buprelab, Labiana Life Sciences S.A. Terrasa, Barcelona, Spain) was added if the pain score was >5. Ciprofloxacin (Ciloxan Eye Drops 0.3%, Alcon Laboratories Inc., Geneva, Switzerland) and tobramycin (Tobrex Sterile Ophthalmic Solution 0.3%, Alcon Laboratories Inc., Geneva, Switzerland) eye drops were administered to the operated eye four times daily for three weeks. Rabbits were monitored twice daily for 7 days, then daily until suture removal at three weeks. Syringe feeding with a rabbit feeding formula (Critical care, Oxbow Enterprises, Inc., Omaha, NE, USA) 15 mL/kg per os 3 times a day was performed as needed if dysorexia or decreased fecal output were noted.

### 2.7. Postoperative Evaluation

#### 2.7.1. Follow-Up Assessments

Corneal and blepharorrhaphy suture removal was performed under sedation (see protocol above), three weeks after surgery, and corneas were followed for 4 months. Postoperative evaluations were conducted at weeks 3, 10, and 16. Each evaluation included comprehensive ophthalmic examinations (comprising slit-lamp evaluation, intraocular pressure measurement, Schirmer test, and fluorescein staining) scoring using the modified McDonald–Shadduck scale, and esthesiometry, as described in the preoperative evaluation. Optical coherence tomography (OCT) and in vivo confocal microscopy were also performed under sedation at each follow-up week (see protocol above). All these examinations were used to assess the biointegration of the implants.

#### 2.7.2. Histopathological Analysis

At 3, 10, and 16 weeks, one rabbit from each group was randomly selected and euthanized under anesthesia (see protocol above) by administering an intracardiac injection of 1.5 mL pentobarbital (Euthanyl^®^ 240 mg/mL, BIMEDA-MTC Animal Health INC., ON, Canada). Eyes were enucleated, formalin-fixed, paraffin-embedded, sectioned (3 uM), and stained with hematoxylin–phloxine–saffron (HPS) for histopathologic examination.

#### 2.7.3. Statistical Analysis

To assess the effect of treatment on individual weight, corneal transparency, corneal thickness, and corneal sensitivity over time, linear mixed models (LMMs) were employed. Individual identity was included as a random factor within the LMMs to account for potential pseudo-replication bias. The normality condition of the residuals was checked visually and via Shapiro–Wilks tests. In order to respect the normality assumption, the Schirmer test values were log10 transformed. Model outcomes were reported using likelihood ratio tests (LRTs). Given that the interaction between corneal graft type and follow-up week had a statistically significant effect on each of the dependent variables mentioned, post hoc tests were performed and corrected for multiple comparisons using the Benjamini–Hochberg method. All statistics were carried out with R software version 4.3.1 (R Core Team (2023) R: A Language and Environment for Statistical Computing. R Foundation for Statistical Computing, Vienna. Available online: https://www.R-project.org/, accessed on 22 April 2024). *p*-values less than 0.05 were considered statistically significant.

## 3. Results

### 3.1. Ophthalmic Examination

The statistical tests conducted indicate that the evolution of individual weight over time is similar across the different treatments, suggesting that the growth of the rabbits was not differentially impacted by the treatment used (*p* = 0.237). None of the rabbits lost weight during the conducted experiments.

It should be noted that the cornea of the first operated rabbit perforated during the surgery, but was nevertheless implanted with a solid CAH (rabbit 1). The patch was successful, and the eye remained intact.

By week 3, when the sutures were removed, complete corneal re-epithelialization was observed in two out of three rabbits in the allograft group and solid CAH implants group. Incomplete re-epithelialization, indicated by positive fluorescein tests, was noted in the three rabbits of the liquid CAH-grafted group, and one each of the solid CAH implant and allograft groups. However, by week 10, all rabbits were completely re-epithelialized, with negative fluorescein tests persisting through the end of the follow-up. Macroscopic evaluation showed successful integration of 8 out of 9 implants into the corneas. Only one implant in the solid CAH group (rabbit 7) was not totally integrated and partially detached during suture removal. The other grafts remained integrated without evidence of wound dehiscence or extrusion during follow-up.

Schirmer test values and intraocular pressure remained within normal ranges throughout the study, and the Tyndall effect was consistently negative [30,31]. There were also no signs of corneal edema, immune rejection, ocular toxicity, or infection. Pericorneal inflammation, assessed by chemosis and conjunctival hyperemia scoring, was present in all rabbits at week 3 but resolved by week 10.

**Figure 1:**
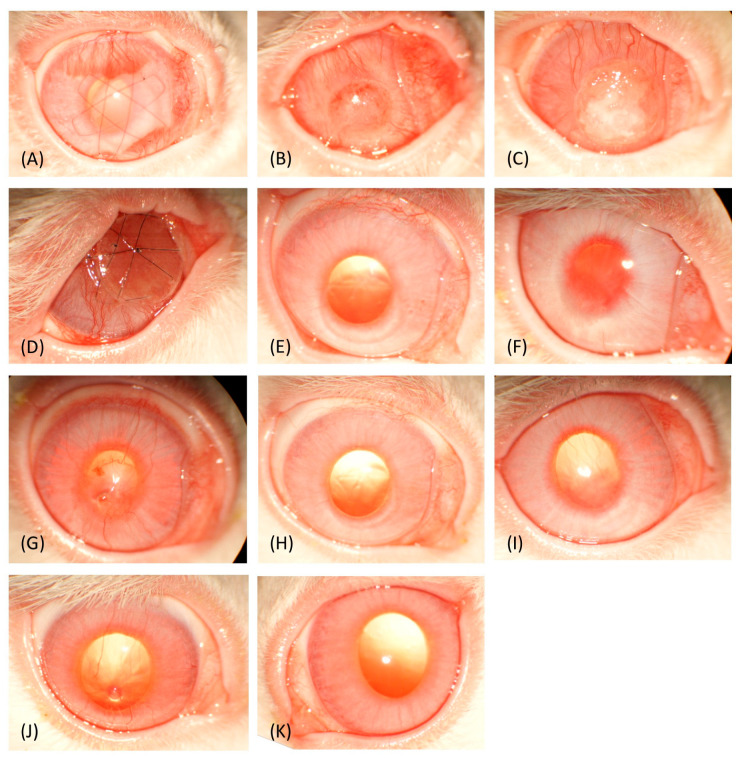
Macroscopic appearance of rabbit eyes during follow-up. Week 3 follow-up: allograft group (**A**), solid collagen group (**B**), liquid collagen group (**C**), rabbit 7 (**D**). Week 10 follow-up: allograft group (**E**), solid collagen group (**F**), liquid collagen group (**G**). Week 16 follow-up: allograft group (**H**), solid collagen group (**I**), liquid collagen group (**J**). Control group: (**K**).

Preoperatively, transparency scores indicated no significant difference in corneal transparency between groups (*p* > 0.05). Throughout the postoperative follow-up, transparency scores of the treated groups remained significantly lower than those of the unoperated control eyes (*p* < 0.05). The corneal transparency was assessed at all follow-up stages, revealing opacities in each group by week 3. Three weeks’ post-operation, the allografts exhibited the highest transparency scores, followed by the solid CAH and liquid CAH implants, with statistically significant differences among the three groups (*p* ≤ 0.001). Slit lamp examination showed dense, superficial, arborized neovascularization in the allografts, slightly penetrating the graft zone from ventral and dorsal regions (Figure 1A). The central graft zone, free of vascular invasion, maintained good transparency. The solid CAH implants exhibited superficial, diffuse neovascularization around the limbus, with central anterior stromal opacity over the graft zone (Figure 1B). The liquid CAH implants presented similar neovascularization with larger caliber vessels and thick, non-transparent, heterogeneous white material over the graft zone, affecting the corneal shape, which was no longer smoothly convex (Figure 1C).

However, by weeks 10 and 16, transparency scores among the treated groups were no longer significantly different (*p* > 0.05). At week 10, all groups showed some improvement, with slight transparency loss in less than 25% of the cornea and no signs of inflammation. In the allograft group, slit lamp examination revealed slight anterior stromal opacity at the incision site with a few ghost vessels, while the remainder of the graft zone remained transparent (Figure 1E). The solid CAH and liquid CAH implants showed satisfactory overall transparency, with few fine corneal vessels demonstrating significant regression in neovascularization across all groups (Figure 1F,G). However, central corneal granulomas (~1 mm diameter), with slight anterior stromal opacity around them, developed in three rabbits: two from the liquid CAH and one from the solid CAH group.

By week 16, all groups showed minimal transparency loss (<10% of the cornea). The allografts had persistent slight anterior stromal opacity at the incision site but maintained clear transparency elsewhere without vascularization (Figure 1H). Both the solid and liquid CAH implants retained a few fine corneal vessels (Figure 1I,J). The remaining rabbit with liquid CAH still had a previously observed granuloma, stable in size. Corneal transparency was complete except for the granuloma in the liquid CAH and slight central anterior stromal opacity over a few millimeters in the solid CAH.

### 3.2. Esthesiometry

**Figure 2:**
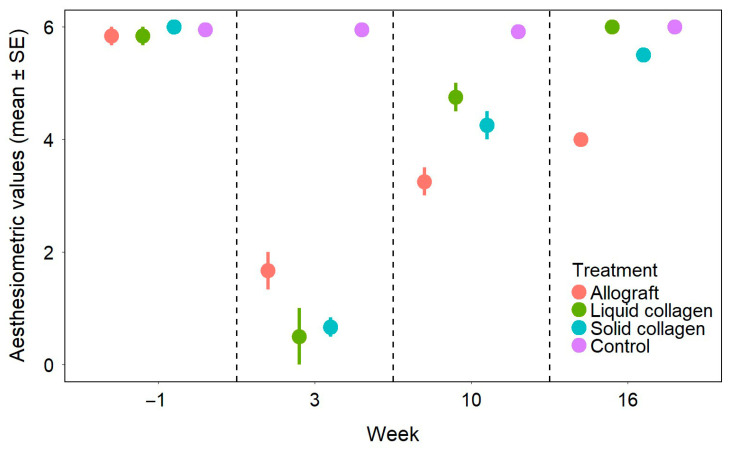
Esthesiometric values of the different groups over time. Data points represent group means, and vertical bars indicate the standard error (SE).

Preoperatively, esthesiometric values (Figure 2) showed no significant differences (*p* > 0.937) between the treated groups (allografts, solid CAH, liquid CAH) and the control group (non-operated eyes). At weeks 3 and 10, treated eyes in all groups had significantly lower esthesiometric values than controls (*p* < 0.001). At week 10, significant differences were observed among treated groups: the allograft group had lower values than the solid CAH and liquid CAH groups (*p* < 0.005). By week 16, this trend persisted, as the allograft group continued to have significantly lower values than the two other treated groups (*p* ≤ 0.005), whereas no significant differences were observed among all the other groups (*p* > 0.05).

### 3.3. Optical Coherence Tomography

**Figure 3:**
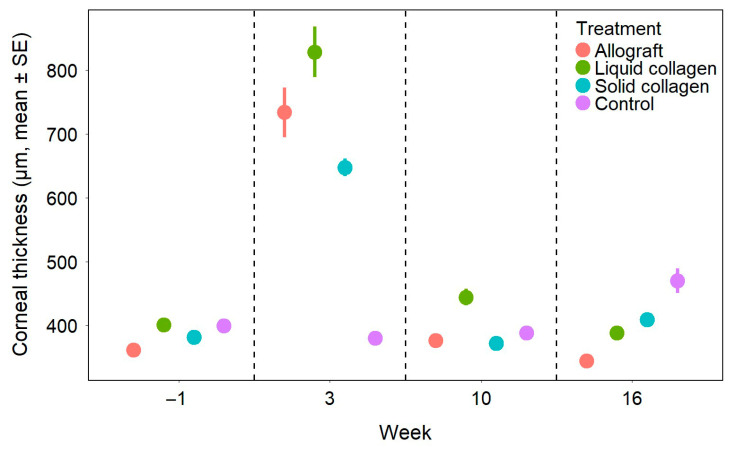
Corneal thickness of the different groups over time. Data points represent group means, and vertical bars indicate the standard error (SE).

**Figure 4:**
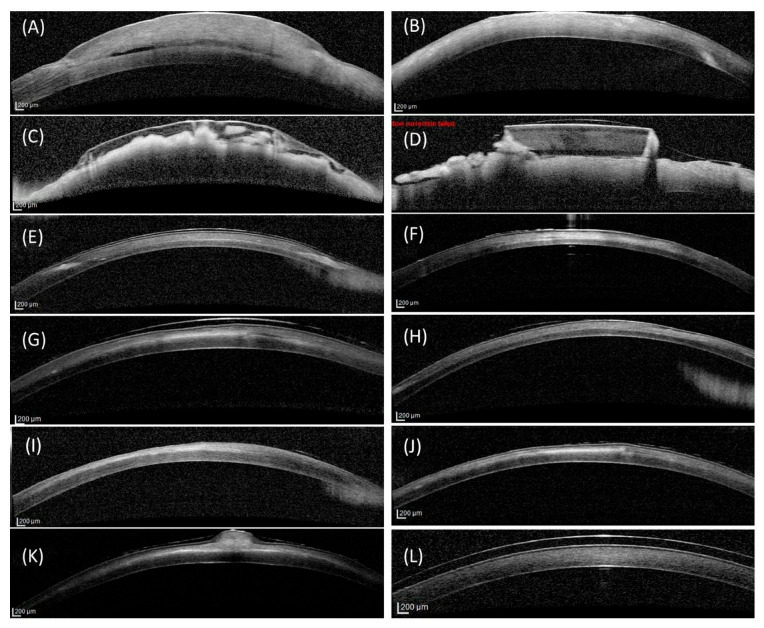
Optical coherence tomography (OCT) appearance of rabbit corneas during follow-up. Week 3 follow-up: allograft group (**A**), solid collagen group (**B**), liquid collagen group (**C**), rabbit 7 (**D**). Week 10 follow-up: allograft group (**E**), solid collagen group (**F**), liquid collagen group (**G**). Week 16 follow-up: allograft group (**H**), solid collagen group (**I**), liquid collagen group (**J**,**K**). Control group: (**L**).

Preoperatively, corneal thickness (Figure 3) was not significantly different among the groups (*p* > 0.05). At week 3 postoperatively, corneal thickness increased significantly in all treated groups compared to the control group (*p* < 0.001), with the allografts and liquid CAH exhibiting greater thickness than the solid CAH (*p* < 0.001). Corneas that received allografts and solid CAH had smooth surfaces and were seamlessly integrated into the host tissue except for Rabbit 7 (solid CAH group). The delineation between the grafted and native zones was visible in some sections as a thin dark band in the stroma in the allograft group (Figure 4A), and the anterior stromal part and epithelium of the grafted zone appeared slightly hyperintense in the solid CAH group (Figure 4B). Rabbit 7’s graft failed to integrate (Figure 1D and Figure 4D). In the liquid CAH group, the corneas exhibited significant thickening with an irregular, heterogeneous surface and a stroma with areas of varying reflectance, including hyperintense and hypointense regions (Figure 4C).

By week 10, corneal thickness was no longer statistically different among the treated and control groups (*p* > 0.05). All corneal layers closely resembled those of a normal cornea morphologically, despite the presence of two hyperintense areas in the anterior stroma of the allografts that were consistent with the haze observed at the incision site and likely represent scar tissue (Figure 4E) and a slightly hyperintense grafted zone in the solid CAH group (Figure 4F). In the liquid CAH group, the corneas showed marked improvement, with normal epithelium and endothelium (Figure 4G). However, the stroma remained slightly heterogeneous, with hyperintense and hypointense areas and occasional thickening that persisted until week 16 (Figure 4J). By week 16, the regenerated liquid CAH neocornea showed an almost normal appearance, though OCT images still revealed a persistent granuloma (Figure 4K).

In rabbits with granulomas, OCT depicted these as thickenings with reflectance similar to the stroma, disrupting epithelial continuity. By week 16, the corneas in the allograft and liquid CAH groups were significantly thinner than those in the solid CAH group and untreated contralateral corneas (*p* < 0.05). However, these differences were not large enough to be clinically relevant. The OCT appearances of the allografted and solid CAH corneas were very similar to that of the control group (Figure 4H,I,L).

### 3.4. Confocal Microscopy

**Figure 5:**
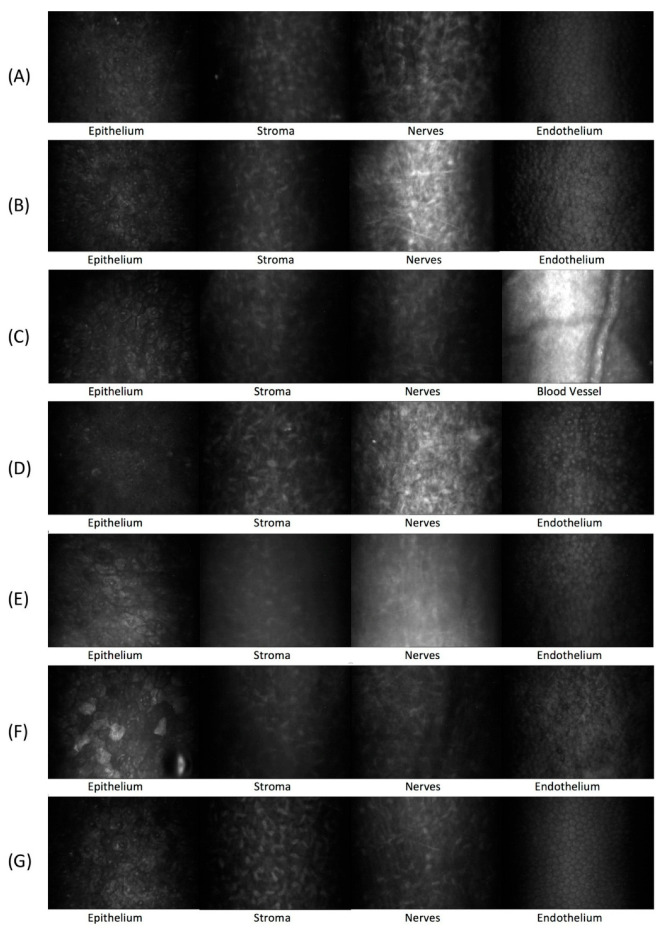
Confocal microscopy appearance of rabbit corneas during follow-up. Week 10 follow-up: allograft group (**A**), solid collagen group (**B**), liquid collagen group (**C**). Week 16 follow-up: allograft group (**D**), solid collagen group (**E**), liquid collagen group (**F**). Control group: (**G**).

At week 3, during the early healing phase, a high cell density was observed in the cornea due to the invasion of keratocytes and inflammatory cells into the grafts post-operatively, leading to haze that obstructed the visualization of corneal layers by IVCM. By week 10, these cells likely ceased migrating and began to become quiescent, resulting in the clearing of haze from the stroma and improving the visualization of corneal layers and keratocytes. At this point, IVCM revealed complete re-epithelialization in all groups, with the epithelium remaining stable through the final follow-up. The allografted and solid CAH implanted corneas had anterior stromal nerves and a normal endothelium by week 10 (Figure 5A,B). In the liquid CAH group, the stroma remained hyper-reflective with active blood vessels at this time, partially hindering visualization of corneal nerves and endothelium (Figure 5C).

By week 16, all groups exhibited normal epithelium and endothelium, stromal corneal nerves, and no active blood vessels. The overall appearance was similar to that of the control untreated contralateral corneas (Figure 5D–G).

### 3.5. Histology

**Figure 6:**
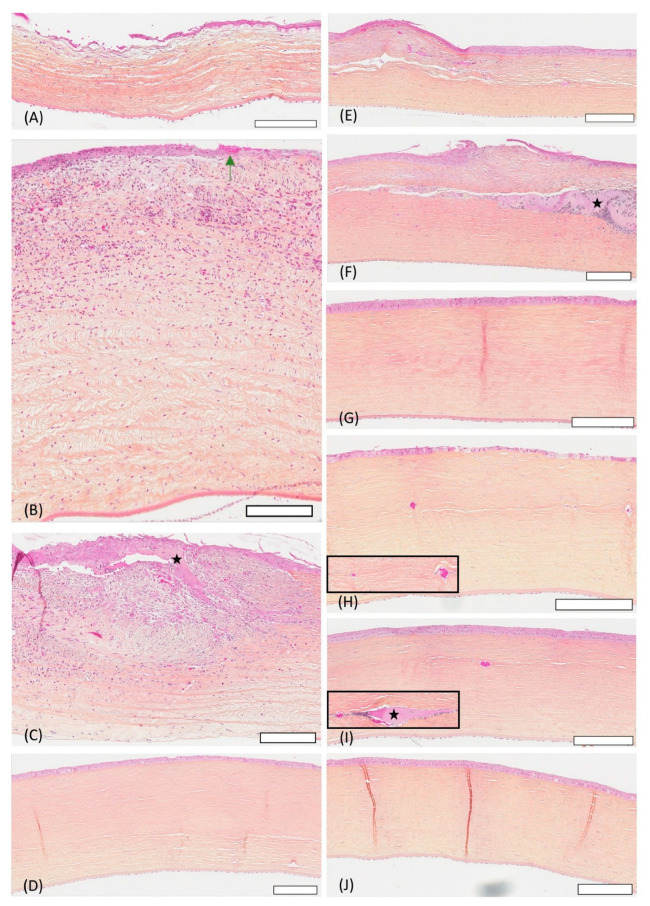
Histological appearance of rabbit corneas during follow-up at low magnification. Bar = 200 μm. Week 3 follow-up: allograft group (**A**), solid collagen group (**B**), liquid collagen group (**C**). Week 10 follow-up: allograft group (**D**), solid collagen group (**E**), liquid collagen group (**F**). Week 16 follow-up: allograft group (**G**), solid collagen group (**H**), liquid collagen group (**I**). Control group: (**J**). Black stars indicate liquid CAH remnants and the green arrow indicate fibrin.

**Figure 7:**
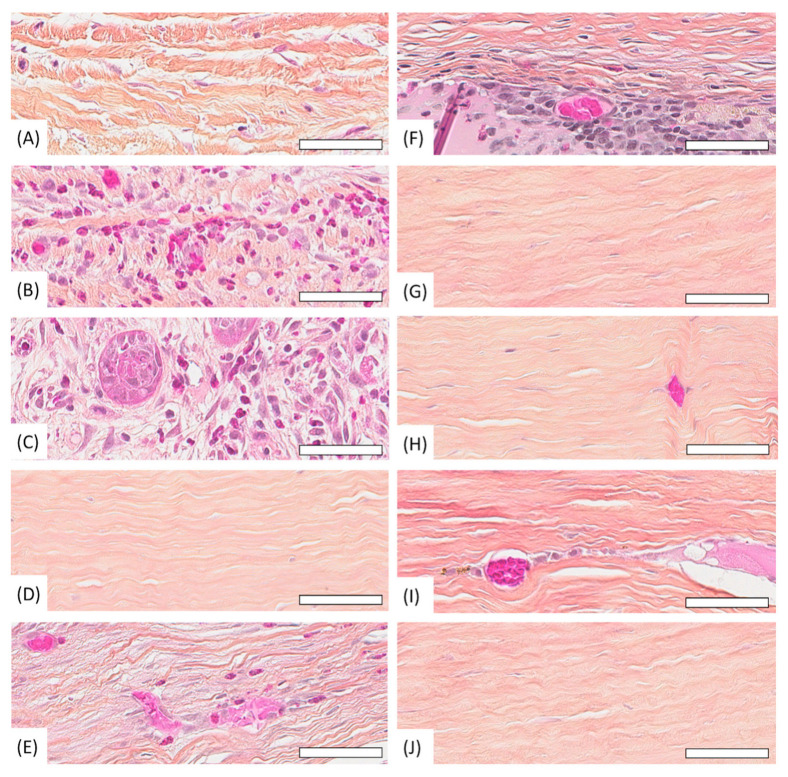
Histological appearance of rabbit corneas during follow-up at high magnification. Bar = 50 μm. Week 3 follow-up: allograft group (**A**), solid collagen group (**B**), liquid collagen group (**C**). Week 10 follow-up: allograft group (**D**), solid collagen group (**E**), liquid collagen group (**F**). Week 16 follow-up: allograft group (**G**), solid collagen group (**H**), liquid collagen group (**I**). Control group: (**J**).

At week 3, rabbits 6, 7, and 9 were euthanized for histological examination. Allografted rabbit 9 had defective re-epithelialization with sparse and edematous basal cells, marked stromal edema, and moderate inflammation (macrophages, heterophils), but no blood vessels (Figure 6A and Figure 7A). Rabbit 7, implanted with solid CAH, had fibrin replacing the absent epithelium at the graft site, notable inflammation (heterophils, macrophages, lymphocytes), and blood vessels, particularly in the anterior stroma, along with slight posterior stromal edema. The cornea was considerably thickened (Figure 6B and Figure 7B). Rabbit 6, which received a liquid CAH, showed partial and disorganized re-epithelialization, extensive stromal inflammation (lymphocytes, heterophils, macrophages), blood vessels, and epithelial cell islands within the stroma. The anterior stroma lacked organization, and eosinophilic material indicated the presence of still cell-free liquid CAH gel (Figure 6C and Figure 7C).

At week 10, rabbits 2, 4, and 8 were euthanized. Allografted rabbit 8 showed an almost normal corneal morphology, with just a few coiled and compact collagen lamellae and no blood vessels or inflammation (Figure 6D and Figure 7D). Rabbit 2, implanted with a solid CAH, presented a neocornea with a distinct epithelium and stroma. However, it still exhibited a 0.5 mm central lesion characterized by an increased number of inflammatory cells, mild anterior stromal edema, numerous keratocytes, and a few blood vessels (Figure 6E and Figure 7E). Liquid CAH-treated rabbit 4 had an epithelium with variable thickness and local disorganization, but the stromal collagen lamellae maintained normal structure and orientation. Some blood vessels remained in the stroma, along with prominent liquid CAH remnants accompanied by inflammatory cells, keratocytes, and anterior stromal edema (Figure 6F and Figure 7F).

At week 16, rabbits 1, 3, and 5 were euthanized. At this point, histological examination demonstrated the ability of all three graft types to reform a structurally native-like neocornea. All groups exhibited a stratified epithelium, a stroma with predominantly well-aligned collagen lamellae, keratocytes oriented parallel to the stromal collagen lamellae, and a normal endothelium. No signs of inflammation were observed in the allografts or solid CAH neocorneas, although rare vessels persisted in the latter (Figure 6G,H and Figure 7G,H). Rabbit 5, which received liquid CAH, showed slight anterior stromal edema in a few areas, a small number of remaining blood vessels, and rare traces of liquid CAH remnants with surrounding macrophages (Figure 6I and Figure 7I).

## 4. Discussion

This study evaluated corneal healing following deep keratectomy in rabbits using three different corneal graft materials. All materials achieved complete re-epithelialization by 10 weeks postoperatively. Given that no fluorescein tests were conducted between weeks 3 and 10, re-epithelialization may have occurred earlier, but this cannot be confirmed. In a study by Simpson et al. using CLP-PEG-MPC implants on mini-pig corneas after alkali burn and keratectomy, re-epithelialization was completed within 7 weeks [21]. A formulation of liquid collagen similar to ours, tested on mini-pigs and cats, allowed for complete re-epithelialization within one month in both species [23,24]. One of these studies also tested this product on rabbits after corneal perforation, but the re-epithelialization time was not evaluated. In our study, two out of three rabbits that received solid grafts (allografts and solid CAH) re-epithelialized by week 3. However, all three rabbits with liquid CAH had re-epithelialization defects at this stage, suggesting that they have a slightly longer healing time with this scaffold in rabbits. This could be explained by the fact that corneal curvature was not restored by week 3 in this group. Macroscopic examination and OCT revealed surface irregularities at this stage, likely delaying epithelialization.

The corneal stroma has a precise structure consisting of a matrix of parallel collagen lamellae and fibrils, with keratocytes being the only cells normally present within the stroma [32]. Histological examinations allowed us to visualize the neo-stroma structure formed after graft implantation and show that it closely resembled that of a native cornea in all groups by 16 weeks postoperatively. The collagen lamellae were well reformed, mostly linear and aligned, keratocytes were present in normal quantities, edema had resolved, and only a few rare blood vessels were observed. OCT measurements indicated significant corneal stroma thickening in all groups at week 3, likely due to postoperative inflammation and inflammatory cell infiltration associated with corneal edema. However, our analyses showed a return to corneal thickness similar to control corneas by week 10. The statistically significant differences in corneal thickness observed at week 16 between groups (untreated control group and solid CAH group > allograft and liquid CAH groups) are probably due to the very small sample size at this stage (only one individual per treated group) and are not clinically significant. The endothelium was not altered during our experiments and maintained its structure and function over time, with no apparent sign of toxicity from the implanted materials.

Corneal transparency and sensitivity are two major functions that allow the cornea to perform its role while protecting against external attacks [33]. In our study, corneal transparency was evaluated macroscopically and scored. At 16 weeks postoperatively, transparency scores were not significantly different among the three treated groups but remained slightly lower than the control group, indicating that transparency was not yet complete at this stage. Since we only followed the rabbits for 16 weeks and transparency continued to improve during the follow-up, this improvement may have continued, but a longer study would be necessary to evaluate the final corneal appearance. It should also be noted that currently available options in veterinary medicine generally result in poorer final transparency compared to allografts. Corneo-conjunctival transpositions tend to become pigmented within months after surgery, while conjunctival grafts and biomaterials derived from other tissues (such as amniotic membrane, intestinal submucosa, or pericardium) often induce significant corneal fibrosis, leading to a noticeable postoperative corneal scar [34,35,36].

The cornea is the most densely innervated organ in the body, and any damage to its nerves can result in loss of function, loss of transparency, and pain [37]. The mechanisms of nerve regeneration within the cornea are complex and not fully understood. Several structures are involved in regulating nerve growth, including epithelial cells, keratocytes, and myofibroblasts. It has been shown that the latter, when activated following corneal injury, inhibit corneal nerve growth [37,38]. ICVM revealed the presence of nerve fibers within the anterior stroma of the grafted area in all groups at week 16, showing that 4 months were sufficient to at least partially regenerate the corneal nerve network. Esthesiometry showed a significant deterioration in corneal sensitivity in all groups at week 3 due to nerve damage caused by surgery. Sensitivity gradually improved and returned to control group levels in the synthetic collagen groups by 16 weeks, indicating effective nerve regeneration within the graft in these groups. In contrast, the allograft group showed persistently reduced sensitivity at week 16, despite gradual improvement over time. This delayed recovery may be related to keratocyte activation, which likely led to their differentiation into myofibroblasts shortly after transplantation and may have hindered nerve regrowth. The synthetic collagen implants, being acellular, lacked such activated cells initially, potentially allowing faster nerve regeneration.

Inflammation is a significant factor in corneal healing, as it can lead to scar formation that impairs vision. We evaluated inflammation histologically and macroscopically using a scoring system. Histologically, inflammation was slightly more pronounced in the liquid CAH group throughout the follow-up compared to the other treated groups, although significant improvement was observed in each follow-up week. By week 16, a few inflammatory cells persisted in the stroma of the liquid CAH group, whereas none were observed in the other groups.

We chose not to use corticosteroids postoperatively to observe the natural healing process. No rejection was observed, and both biosynthetic implants and allografts were well-tolerated by the host corneas, causing minimal and transient inflammation despite the absence of steroids. The Tyndall effect remained negative throughout the follow-up, showing the absence of intra-ocular inflammation. Neovascularization, which was significant in the treated groups at week 3, subsequently decreased or disappeared. Additionally, slight conjunctival hyperemia and chemosis were observed at week 3, likely due in part to the blepharorrhaphy performed to protect the cornea postoperatively, which resolved quickly. Blepharorrhaphy was performed on the operated eyes in our study to protect the surgical site while avoiding the use of an Elizabethan collar, which would have affected animal welfare. In human patients or other animal species, this precaution may not be necessary. These results align with other studies testing similar scaffolds, where postoperative ocular inflammation was also minimal [21,23,24].

Two complications were observed during the study. First, one of the solid CAH implants (rabbit 7) did not integrate properly into the cornea and detached upon suture removal. We hypothesize that the keratectomy in this rabbit was not deep enough, causing the implant to protrude significantly from the corneal curvature during insertion, and the mattress sutures were not tight enough to prevent micro-slippage in the anteroposterior plane. This likely caused friction between the implant and native stroma, resulting in chronic inflammation, as evidenced by the large number of inflammatory cells seen histologically. Chronic corneal inflammation increases corneal thickness through inflammatory cell infiltration and associated edema, which could also explain the corneal thickening observed in rabbit 7 at histology and OCT at week 3.

Additionally, a small granuloma, approximately 1 mm in diameter, appeared in the center of the cornea in week 10 in one rabbit from the solid and two rabbits from the liquid CAH groups. Rabbit 5 with a liquid CAH implant showed formation of a granuloma at week 10, which persisted through week 16. It is possible that one component of the two synthetic collagens triggered granuloma formation. PEG, in particular, with its pro-inflammatory properties, could have played a role. This phenomenon has not been previously reported in studies using CLP-PEG, whether in solid or liquid form [17,21,23,24]. However, in these previous studies, histopathological examinations were only performed after 6, 9, or 12 months. Furthermore, it is important to note that only 3 rabbits had been implanted with each CAH. The development of these granulomas after corneal surgery strongly suggested pyogenic granulomas, a rare entity that can develop following abnormal healing [39]. Unfortunately, we could not observe them histologically to confirm the diagnosis. A 1995 article reported their appearance in 10 rabbits with limbal stem cell deficiency following keratectomy, without providing a precise explanation for the phenomenon [40]. To our knowledge, no further mentions of granulomas occurring after keratectomy in rabbits have been made since. Nonetheless, there is a clinical case report of foreign body granuloma formation in a patient injected with PEG as a tissue filler [41]. Given the limited number of animals in our pilot study, larger animal groups are needed to determine whether these granulomas are indeed related to PEG or other components of the CAH or if environmental or genetic factors played a role. Additionally, these granulomas might not have developed with postoperative corticosteroid treatment, as was employed in previous studies [16,22,24,26].

No significant differences were observed in any assessed parameters among the control eyes of the three groups, nor were there any effects on the weight of the rabbits or the clinical examinations conducted during the follow-up weeks, indicating no systemic effects of the tested products. Temporary episodes of anorexia or dysorexia were noted in some animals following surgery or sedation for examinations. However, appetite returned to normal within a few days. These observations were attributed to the effects of anesthesia on gastrointestinal transit rather than discomfort caused by the grafts.

As this is a pilot study, the most significant limitation is the small sample size in each treatment group, which does not allow for definitive conclusions on the efficacy of the tested products. However, as previously mentioned, this pilot study aimed to observe and describe corneal responses to the three treatments in a limited number of individuals. Another limitation is the health status of the cornea at the time of transplantation. No corneal lesions were induced before the biomaterial grafts, which does not accurately reflect the typical condition of treated corneas in clinical practice.

The use of these biomaterials offers the advantage of reducing surgical time, as the solid implant is ready for deployment without any preparation. In cases where general anesthesia is contraindicated—such as in elderly or unstable animals—the liquid implant could serve as a corneal seal for stromal defects or perforations, providing immediate structural support and promoting effective corneal regeneration in the weeks following application, without the need for suturing a biomaterial [23,24]. This product could therefore be particularly valuable in veterinary clinical practice, offering a simple and time-efficient option for non-specialist veterinarians to manage deep corneal ulcers or perforations, which are extremely common in companion animals.

## 5. Conclusions

We showed that deep keratectomy followed by the application of collagen-like peptide substitutes or allografts in rabbits results in a fully re-epithelialized, transparent, non-inflamed, and well-innervated corneal stroma within 16 weeks in our sample. Given their performance in recent publications and in this pilot study, both forms of collagen-analog hydrogels (solid and liquid implants) appear to be effective alternatives to current transplantation techniques. They have also demonstrated their ability to regenerate corneas that are stably and seamlessly integrated into the host tissue without any risk of disease transmission [21,23,24,42]. However, evidence of possible PEG-induced granulomas suggests that removing this component would be beneficial, at least in the liquid formulation that requires in situ gelation. This study is part of a larger series of investigations and will serve as a basis for designing larger-scale research, helping to draw more definitive conclusions about the long-term therapeutic potential of these substitutes, particularly in pathologic corneas. Their development in veterinary ophthalmology may offer a promising alternative to existing biomaterials, given their potential to achieve a good corneal transparency and reduce postoperative scarring. This could lead to a better visual prognosis for severe corneal ulcers or perforations in companion animals.

## Data Availability

The original contributions presented in this study are included in the article. Further inquiries can be directed to the corresponding authors.
